# *In vitro* antigen presenting cell-derived IL-10 and IL-6 correlate with *Trichuris muris* isolate-specific survival

**DOI:** 10.1111/j.1365-3024.2008.01088.x

**Published:** 2009-03

**Authors:** R D'Elia, K J Else

**Affiliations:** Faculty of Life Sciences, University of ManchesterManchester, UK

**Keywords:** *macrophages*, *dendritic cells*, Trichuris muris, *IL-6 and IL-10*

## Abstract

*Trichuris muris*, the mouse whipworm, is used as a laboratory model of the human parasite *T. trichiura*. Three laboratory isolates of *T. muris* exist — the E, J and S isolates. Previous data have shown that the S isolate survives to chronicity in C57BL/6 mice unlike the E and J isolates, which are expelled. The ability of the S isolate to persist is thought to be due to it secreting unique excretory/secretory antigens, which interact with APCs such that protective T cell responses do not develop. To determine whether APCs respond differently to E/S antigens from the three isolates we cultured isolate-specific E/S with bone marrow-derived macrophages (BMMΦ) and dendritic cells (BMDCs) *in vitro*. Markers of co-stimulation and levels of MHC-II were analysed by FACS and cytokine levels in supernatants quantified. E/S antigens from the S isolate consistently stimulated significantly higher levels of IL-10 and IL-6 from both macrophages (F4/80^+^CD11b^+^CD11c^−^) and dendritic cells (CD11c^+^CD11b^+^F4/80^−^) compared to J and E isolate E/S. If these *in vitro* differences in APC-derived cytokines, particularly IL-10, are biologically significant *in vivo*, they may contribute to the S isolate survival, by creating a regulatory cytokine environment in which protective immune responses are less effective.

## INTRODUCTION

*Trichuris muris* is a natural helminth of mice and serves as a relevant model for the infection of humans with the gastrointestinal nematode parasite *T. trichiura* (1). *Trichuris trichiura* is believed to infect around 1 billion people worldwide.

Through the use of this laboratory model, our understanding of immunity to intestinal parasites has significantly increased. Thus it is now well-established that resistance to *T. muris* is associated with a dominant T helper (Th) 2 response and susceptibility a Th1 response (2,3).

The majority of *T. muris* studies focus on one particular isolate, the Edinburgh (E) isolate, however, two other isolates exist, the Japan (J) and Sobreda (S) isolates. Remarkably, the S isolate of *T. muris* is able to survive to chronicity in the C57BL/6 mouse; whereas the other two isolates are readily expelled from the same strain of mouse (4–7). Little is known about the underlying mechanisms associated with S isolate survival. Previous published data from our laboratory suggested that the differences in expulsion kinetics *in vivo* could be associated with a difference in protein make-up of the excretory/secretory products (E/S) between the isolates (7).

E/S products from helminths are antigenic but are also believed to have immunomodulatory properties, and this may explain why helminths protect against the development of autoimmunity and allergy (8–10).

E/S products from *Ascaris suum* and *Nippostrongylus brasiliensis* have both been shown to inhibit lung inflammation by suppressing levels of IL-4 and IL-5 (11,12). E/S products are a very heterogeneous mix; research therefore, has started to focus on specific proteins within the E/S. Of particular interest is the immunomodulatory properties of a 62 kDa protein isolated from *Acanthocheilonema viteae*(13). ES-62 is the major secreted glycoprotein of the rodent filarial nematode *A. viteae* and contains the unusual post-translational modification of phosphorylcholine (PC) addition (14–16). ES-62 has been shown to have a wide range of immunomodulatory properties and has been shown to be effective as a treatment for collagen-induced arthritis (17). It is believed that E/S from helminths and ES-62 in particular can modulate the immune response in several ways including via interactions with B cells, macrophages and dendritic cells (18,19). Data suggest that exposure of macrophages to ES-62 makes them unable to produce Th1/proinflammatory cytokines (20). In addition ES-62 drives the maturation of DCs and thus promotes the differentiation of naïve CD4+ T cells towards a Th2 phenotype (21). The ability of E/S to alter innate cell function is not uncommon and has been demonstrated for a number of other parasites (22,23). Interestingly both differentiated and undifferentiated intestinal pig epithelial cells predominantly produce IL-10 and IL-6 following stimulation with *T. suis*E/S (24).

The ability of E/S to alter the biology of macrophages and dendritic cells (DC) is of particular interest given their involvement in the induction and maintenance of adaptive immune responses. Thus the response of DCs and/or macrophages to the S isolate E/S antigens may underlie the subsequent inability of the host to mount a protective Th2 response.

In this study we explore the ability of E, J and S isolate E/S to alter the phenotype and function of bone marrow-derived macrophages (BMMΦ) and bone marrow-derived dendritc cells (BMDCs). Cell surface expression of the co-stimulatory markers CD80, CD86 and CD40 along with MHC class II were analysed by flow cytometry on BMMΦ and BMDCs after exposure to isolate-specific E/S. In addition the cytokine levels secreted by these APCs were analysed by cytokine bead array.

We hypothesize that BMMΦ and or BMDCs would respond differently to S isolate E/S antigens compared to E/S from the E and J isolates at the level of costimulatory markers and/or cytokine production.

## MATERIALS AND METHODS

### Mice

C57BL/6 male mice were obtained from Harlan (Bicester, UK) at 6–8 weeks of age. Mice were specific pathogen-free and maintained in sterile conditions in individually ventilated cages by the Biological Services Facility (BSF), University of Manchester, UK. All work was performed under the regulations of the Home Office Scientific Procedures Act (1986).

### Parasites

The E, J and S isolates of *T. muris*were maintained as previously described (25). Excretory/secretory (E/S) antigen was collected by incubating adult worms in RPMI medium for 4 h. To confirm that the E/S antigen was not contaminated with bacterial LPS or any other endotoxins a Limulus Amebocyte Lysate (LAL) Test Kit (Charles River Endosafe) was used (Levels for endotoxin in all samples of isolate E/S were all below 3 pg/mL). Previous data show that > 300 pg/mL of endotoxin is required to cause a cytokine response (26).

### Bone marrow macrophage culture

BMMΦ were cultured as previously described (26). Briefly bone marrow was recovered and pooled from the femurs of 3–5 naive C57BL/6 mice by flushing the bone with Dulbecco's modified Eagle's medium (DMEM; Invitrogen, Paisley, UK) containing 10% fetal calf serum, 2 mm l-glutamine, 100 units/mL penicillin and 100 ng/mL streptomycin (complete DMEM). The cell suspension was passed through a 21 gauge needle, washed and cultured at 1 × 10^6^/mL in complete DMEM containing 30% L929-conditioned media for 7 days. Macrophage purity was assessed by staining with anti-CD11b-PE and anti-F4/80-FITC (BD Biosciences, Oxford, UK). Greater than 80% of live cells [measured by 7-amino-actinomycin D (Sigma) exclusion] were positive for either or both markers. Less than 1% of cells were positive for CD11c.

### Bone marrow dendritic cell culture

Bone marrow was recovered and pooled from the femurs of 3–5 naïve C57BL/6 mice by flushing the bone with complete medium (RPMI 1640 (Invitrogen, Carlsbad, CA) containing 5% foetal calf serum (FCS), 100 U/mL penicillin and 100 µg/mL streptomycin, 1%l-glutamine, 0·1% MTG (Sigma-Aldrich, St Louis, MO) on ice. The cell suspension was passed through a 21 gauge needle and washed. Cell concentration was determined using a CASY-1 Cell Counter. DC culture followed a previous publication (27). Briefly, following centrifugation the cell pellet was counted and resuspended at 2 × 10^6^ cells/mL in 10 mL of RPMI supplemented with 10% FCS, 100 U/mL Penicillin, 100 ng/mL Streptomycin, 2 mm l-glutamine and 0·5 mL of GM-CSF at 40 ng/mL (GM-CSF-containing supernatants were derived from Ag8 hybridoma cells (28)) and plated in bacteriological Petri dishes. A further 10 mL GM-CSF-containing medium was added after 3 days culture, and replaced after 6 days. After 7 days culture the non-adherent DCs were removed and purity was assessed by staining with anti-CD11b-PE and anti-CD11c-FITC (BD Biosciences, Oxford, UK). Greater than 65% of live cells [measured by (7AAD) 7-amino-actinomycin D (Sigma) exclusion] were positive for either or both markers. Fewer than 1% of cells were positive for F4/80.

### Protocol

The BMMΦ at 1 × 10^6^/mL or BMDC at 1 × 10^6^/mL were added to 12-well plates and cultured for 48 h in media alone – unstimulated (U), 50 µg/mL E isolate E/S (E), 50 µg/mL J isolate E/S (J), 50 µg/mL S isolate E/S (S) or 100 ng Lipopolysaccharide (LPS). The same batch of E/S from each of the three isolates and LPS was used for all four independent BMMΦ and four independent BMDC experiments to minimize any batch to batch differences. Supernatants were assessed using a custom cytometric bead array kit (CBA- BD biosciences) and cells were collected for FACs analysis. Four wells of BMMΦ or BMDC were cultured with each condition. The cells from each of the four wells were combined for FACs analysis, but supernatants were kept separate and analysed individually. This was repeated a further three times.

### Cytokine bead array (CBA)

Supernatants were assessed using a custom cytometric bead array kit (CBA-BD biosciences) for IL-10, IL-12p70, IFN-γ, TGF-β, IL-6, TNF-α, MCP-1 and MIP-1α. Briefly samples were incubated with the combined capture bead cocktail, diluted in capture bead diluent and incubated for 1 h at RT on a digital shaker at 500 r.p.m. Following incubation, samples and beads were mixed with the combined cocktail of PE detection antibodies for the eight cytokines and incubated for 1 h. Sample was then washed and resuspended in FACS buffer. Cytokine concentrations were measured via quantification of PE fluorescence of samples in reference to a standard curve.

### Flow cytometric analysis

BMMΦ and BMDCs were stained as a single suspension in 96 well plates. Cells were stained at a concentration of 1 × 10^6^ cells per 200 µL FACs buffer (0·1% BSA, 0·05% Sodium Azide in PBS). Plates were washed in FACS buffer and spun at 3000 g for 5 min between each staining step and twice before acquisition. Non-specific antibody binding was prevented via incubation at 4°C with 0·25 µg anti-Fc-γ Receptor I/II (CD16/CD32) antibodies (BD Biosciences). Cells were then stained for either CD80-biotin (16-10A1), CD86- biotin (GL1), CD40-biotin (1C10) or MHC class II- biotin (M5/114·15·12) by incubation for 30 min at 4°C. A second antibody step consisted of adding a master mix of CD11b-PE (M1/70), F4/80-FITC (6F12) or CD11c-FITC (HL3), 7AAD and SA-APC for a further 30 min at 4°C in the dark. Relevant isotype controls were added for each antibody to duplicate samples. Cells were finally resuspended in equal quantities of FACS buffer and FACS Fix (2% formalydehyde in PBS). Samples were analysed on FACSCalibur (Becton Dickinson) using CellQuest Pro software (BD Biosciences, Mountain View, CA). Geomean levels were recorded for samples stained for surface markers and isotype control samples to generate a fold-change ratio of geomean surface marker/geomean isotype control.

### Statistics

Cytokine results are expressed as means ± standard deviation (SD). Statistical analysis was performed using the one-way anova with a Bonferroni post-test. Significant differences on graphs are only shown between the isolates for clarity. All statistical tests were performed using GraphPad Prism software (San Diego, CA).

## RESULTS

### Isolate-specific E/S fail to consistently alter BMMΦ cell surface expression of CD80, CD86 and MHC class II

**Figure 1:**
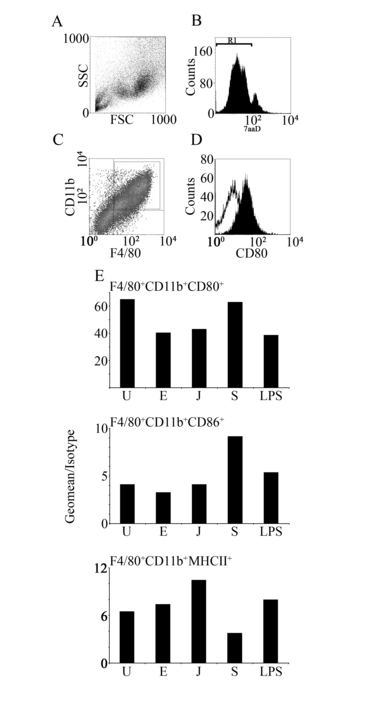
Bone marrow macrophages (BMMΦ) stimulated with parasite specific E/S show no consistent change in co-stimulatory cell surface markers compared to unstimulated levels. Bone marrow was isolated and pooled from 3–5 naïve C57BL/6 mice and cultured into BMMΦ. The BMMΦ were incubated for 48 h, *in vitro* with medium alone – unstimulated (U), 50 µg/mL E isolate E/S (E), 50 µg/mL J isolate E/S (J), 50 µg/mL S isolate E/S (S) or 100 ng Lipopolysaccharide (LPS). The cells were analysed for cell-specific markers F4/80, CD11b, co-stimulatory markers CD80 and CD86 and antigen recognition marker MHCII. A representative scatter plot is shown (a) with analysis performed on cells negative for 7AAD-R1 (b) and double positive for CD11b and F4/80 (c). Levels of CD80, CD86 and MHC class II were then measured on these cells with a representative histogram showing CD80 expression on untreated cells (filled histogram) compared to its relevant isotype control (unfilled histogram) (d). Geomeans for unstimulated, E, J or S isolate antigen and LPS stimulated BMMΦ and relevant isotype controls were calculated and a fold change for Geomean anti cell surface marker antibody/Geomean Isotype control antibody was plotted for CD80, CD86 and MHCII expression on F480^+^CD11b^+^7AAD^−^cells (e). Experiments were performed four times and data shown are a representative experiment.

BMMΦ were cultured with media alone, E isolate E/S, J isolate E/S, S isolate E/S or Lipopolysaccharide (LPS) as a positive control. Following 48 h of culture, cells were stained for cell surface markers associated with a macrophage phenotype F4/80, CD11b and cell viability marker 7AAD in addition to the co-stimulatory markers CD80, CD86 and MHC class II. Dead cells were eliminated from the analysis by grouping on cells negative for 7AAD (Figure 1b). The remaining analysis was then done on cells that were double positive for F4/80 and CD11b (Figure 1c), with the double positive quadrant determined by the isotype control profile (data not shown). The percentage of BMMΦ positive for both F4/80 and CD11b ranged from 60% to 70% for each of the four experiments. In addition separate BMMΦ samples were taken and analysed for the level of CD11c expression. Samples that were F4/80^+^ or CD11b^+^or F4/80^+^CD11b^+^ were all < 1% positive for CD11c (data not shown). Cells that were F4/80^+^CD11b^+^7AAD^−^ were analysed for CD80, CD86 and MHC class II expression.

The level of expression of CD80, CD86 and MHC class II was calculated using the geomean levels and comparing them to their relevant individual isotype controls. An example of CD80 expression on F4/80^+^CD11b^+^7AAD^−^ cells of a positive sample compared to an isotype sample is shown (Figure 1d). The geomean of the sample was divided by the geomean of the isotype controls and is therefore shown as a fold change in Figure 1(e).

No consistent changes in the geomean ratios for CD80, CD86 or MHC class II were seen on BMMΦ that were F4/80^+^CD11b^+^7AAD^−^ (Figure 1e). Examples shown are values that were consistent on two out of the four experiments. On those two occasions the S isolate E/S increased levels of CD80 and CD86 but decreased levels of MHC class II compared to the E isolate and J isolate E/S (Figure 1e), although unstimulated cultures had a geomean ratio for CD80 expression above values of cells cultured with isolate specific E/S and LPS.

### Significantly higher levels of IL-10 and IL-6 are secreted by BMMΦ following stimulation with S isolate E/S compared to E or J isolate E/S

**Figure 2:**
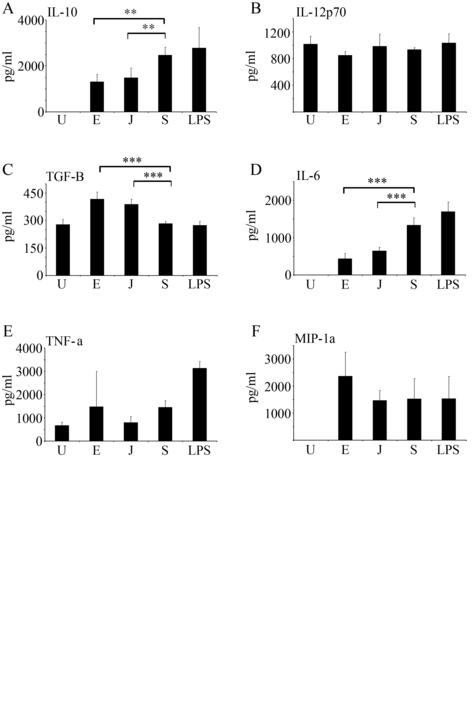
Bone marrow macrophages (BMMΦ) stimulated with S isolate excretory/secretory antigen (E/S) secrete higher levels of IL-10 and IL-6 compared to BMMΦ stimulated with E or J isolate E/S. Bone marrow was isolated and pooled from 3–5 naïve C57BL/6 mice and cultured into BMMΦ. The BMMΦ were incubated for 48 h, *in vitro* with medium alone – unstimulated (U), 50 µg/mL E isolate E/S (E), 50 µg/mL J isolate E/S (J), 50 µg/mL S isolate E/S (S) or 100 ng Lipopolysaccharide (LPS). Levels of IL-10, IL-12p70, TGF-β, IL-6, TNF-α and MIP-1α were measured by CBA; all cytokine data are pg/mL. Values represent the mean ± SD for four wells per treated group. Significant difference between the BMMΦ stimulated with the S isolate E/S antigen and BMMΦ stimulated with the E or J isolate E/S antigens. **P < 0·01 and ***P < 0·001. Experiments were performed four times and data shown are a representative experiment.

Following the culture of BMMΦ for 48 h with medium alone, E isolate E/S, J isolate E/S, S isolate E/S or LPS the supernatants were analysed for a panel of cytokines. S isolate E/S significantly increased levels of IL-10 and IL-6 cytokines in the culture medium compared to E/S from the two other isolates (Figure 2a,d). This significant elevation was consistent in all four experiments with levels of both these cytokines undetectable in the unstimulated controls. In addition, levels of TGF-β were significantly decreased following stimulation with S isolate E/S compared to levels of this cytokine produced by BMMΦ stimulated with E isolate or J isolate E/S (Figure 2c). Levels of IL-12p70, TNF-α and MIP1-α did not differ significantly between the isolates (Figure 2b,e,f, respectively). Further, levels of TNF-α and IL-12p70 secreted by BMMΦ following addition of isolate-specific E/S were not significantly different to unstimulated (medium alone) samples. In contrast, levels of MIP1-α were elevated from unstimulated levels to levels equivalent to the LPS control for all three isolates (Figure 2e). In addition to these cytokines presented, levels of Th2-associated cytokines IL-4, IL-5, IL-9 and IL-13 were analysed and found to be undetectable (data not shown).

### Exposure of BMDCs to isolate E/S results in elevated levels of CD40 expression

BMDCs were cultured for 48 h with isolate-specific E/S or LPS and analysed for the expression of CD80, CD86, CD40 and MHC class II.

**Figure 3:**
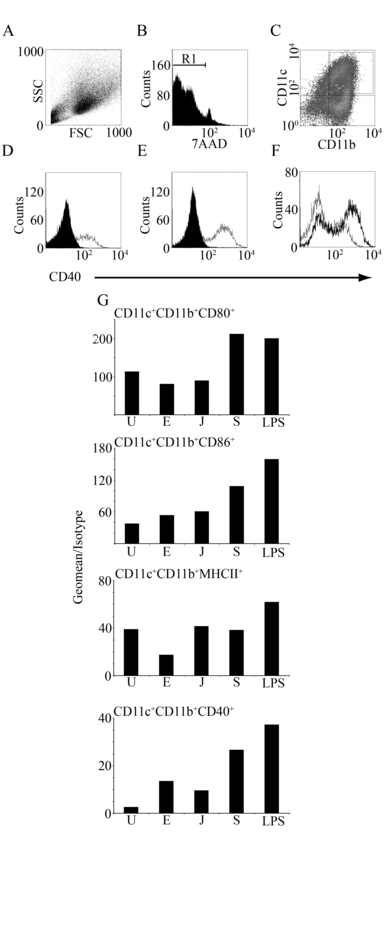
Bone marrow dendritic cells (BMDC) stimulated with isolate E/S show increased levels of CD40 compared to unstimulated levels.Bone marrow was isolated and pooled from 3–5 naïve C57BL/6 mice and cultured into BMDCs. The BMDCs were incubated for 48 h, *in vitro* with media alone – unstimulated (U), 50 µg/mL E isolate E/S (E), 50 µg/mL J isolate E/S (J), 50 µg/mL S isolate E/S (S) or 100 ng Lipopolysaccharide (LPS). The cells were analysed for cell specific markers CD11c, CD11b, co-stimulatory markers CD80, CD86, CD40 and antigen recognition marker MHCII. A representative scatter plot is shown (a) with analysis performed on cells negative for 7AAD-R1 (b) and double positive for CD11c and CD11b (c). Levels of CD80, CD86, CD40 and MHCII were then measured on these cells with representative histograms showing CD40 expression on unstimulated cells (unfilled histogram) compared to its relevant isotype control (filled histogram) (d), LPS treated (unfilled histogram) compared to isotype control (filled histogram) (e) and LPS CD40 profile (dark line) compared to unstimulated CD40 expression profile (grey line) (f). Geomeans for unstimulated, E, J or S isolate antigens and LPS stimulated BMDC and isotype controls were calculated. A fold change ratio for Geomean anti-cell surface marker antibody/Geomean Isotype control antibody were plotted for CD80, CD86, CD40 and MHCII expression on CD11c^+^CD11b^+^7AAD^−^ cells (e). Experiments were performed four times and data shown are a representative experiment.

7AAD^+^ cells were classed as dead and removed from further analysis (Figure 3b). 7AAD negative cells were stained for DC phenotype markers CD11c and CD11b to assess purity of the culture prior to downstream analysis of co-stimulatory markers and MHC class II (Figure 3b,c). The Percentage of CD11c^+^CD11b^+^ cells ranged from 55% to 65% across all four experiments. In addition, separate samples were stained for F4/80 to exclude any macrophage contamination. In all cases cells were F4/80 negative (data not shown). Levels of CD80, CD86, CD40 and MHC class II were assessed individually on cells that were CD11c^+^CD11b^+^7AAD^−^. Levels of cell surface marker expression were measured using the geomean. Geomeans for relevant isotypes were also obtained and used to create a fold-change value of geomean sample/geomean isotype control. Examples of CD40 expression of cells cultured in medium alone with the relevant isotype control for CD40 (Figure 3d), cells cultured with LPS and stained with anti-CD40 and relevant isotype control (Figure 3e) and levels of CD40 expression on unstimulated BMDCs compared to cells exposed to LPS (Figure 3f) are shown.

In two out of four experiments the S isolate E/S increased the levels of CD80 and CD86 expression on CD11c^+^CD11b^+^ 7AAD^−^ cells (Figure 3g). In all four experiments, BMDCs stimulated with either the E, J or S isolate E/S consistently increased levels of CD40 expression compared to medium alone samples (Figure 3g). There was however no consistent difference between the isolates. LPS up-regulated the levels of expression of CD80, CD86, MHC class II and CD40 on BMDCs compared to unstimulated levels in all four experiments.

### Levels of IL-10 and IL-6 secreted by BMDCs were significantly increased following stimulation with S isolate E/S compared to the E or J isolate E/S

**Figure 4:**
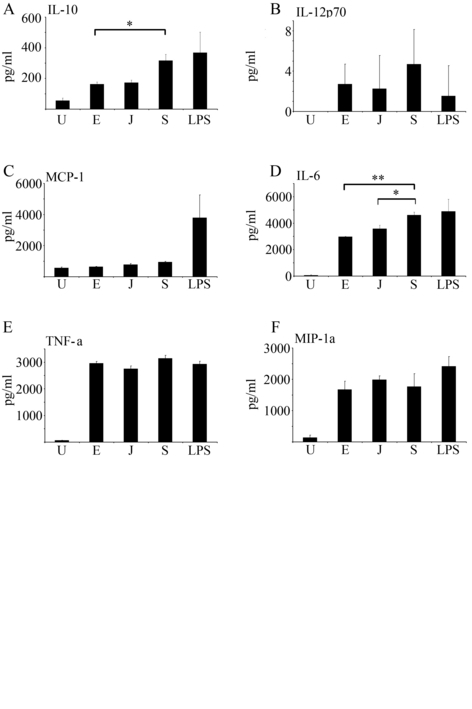
Bone marrow dendritic cells (BMDC) stimulated with S isolate excretory/secretory antigen (E/S) secrete higher levels of IL-10 and IL-6 compared to BMDC stimulated with E or J isolate E/S. Bone marrow was isolated and pooled from 3–5 naïve C57BL/6 mice and cultured into BMDCs. The BMDCs were incubated for 48 h, *in vitro* with medium alone – unstimulated (U), 50 µg/mL E isolate E/S (E), 50 µg/mL J isolate E/S (J), 50 µg/mL S isolate E/S (S) or 100 ng Lipopolysaccharide (LPS). Levels of IL-10, IL-12p70, MCP-1, IL-6, TNF-α and MIP-1α were measured by CBA; all cytokine data are pg/mL. Values represent the mean ± SD for four wells per treated group. *Significant difference between the BMDCs stimulated with the S isolate E/S antigen and BMDCs stimulated with the E or J isolate E/S antigens *P < 0·05, **P < 0·001 and ***P < 0·001. Experiments were performed four times and data shown are a representative experiment.

BMDCs cultured with isolate-specific E/S, LPS or medium alone (unstimulated) were analysed for a panel of cytokines. As seen for BMMΦ, levels of IL-10 and IL-6 secreted by BMDCs were significantly elevated following S isolate stimulation compared to stimulation with E/S from the other two isolates (Figure 4a,d, respectively).

Levels of TNF-α, IL-12p70 and MIP-1α did not vary between isolates, but in all cases, were elevated from unstimulated levels and were equivalent to levels from cells exposed to LPS (Figure 4b,e,f). Unpublished observations from our laboratory have also shown no differences in BMDC-derived TGF-β levels after exposure to the three isolate antigens. In accordance with data presented for BMMΦs, levels of Th2 associated cytokines IL-4, IL-5, IL-9 and IL-13 were analysed and found to be undetectable (data not shown).

## DISCUSSION

In this study we explore the hypothesis that the ability of C57BL/6 mice to mount a protective Th2 immune response to the J and E isolates of *T. muris* but a nonprotective Th1 immune response to the S isolate is due to isolate-specific E/S antigens interacting differently with key APCs.

Little is known about the responses of macrophages or DCs to *T. muris* antigens although it has been reported that BMMΦ from C57BL/6 wild type mice and mannose receptor KO mice, secrete increased levels of IL-6, IL-10, CCL3 (MIP1-α) and CCL9 following stimulation with the E isolate of *T. muris* E/S antigen (26).

Here we culture BMMΦ and BMDCs from naïve C57BL/6 mice with the E/S from the E, J and S isolates of *T. muris* to identify differences in BMMΦ and/or BMDC responses (at the level of cytokine secretion or cell surface marker expression) which may underlie the subsequent documented differences in Th subset responses and thus outcome of infection (5–7). We demonstrate three novel findings:

Firstly BMMΦ cultured with S isolate E/S secrete significantly higher levels of IL-10 and IL-6 compared to BMMΦ stimulated with the E or J isolate E/S. Secondly this phenomenon is not restricted to macrophages with BMDC also secreting elevated levels of IL-10 and IL-6 when stimulated with S isolate E/S compared to stimulation with the other two isolates. And finally, we identify no consistent differences in cell surface expression of co-stimulatory markers and MHC class II on BMMΦ and BMDC after exposure to E, J or S isolate E/S, although BMDCs consistently express increased levels of CD40 after culture with all three types of E/S compared to unstimulated samples.

Helminths have often been termed ‘masters of regulation’ (29), and this ability to regulate has been hypothesized to be due to parasite-derived E/S proteins. Several studies have focused on the immunomodulatory effects of E/S from helminths (19,30) but of particular interest has been a molecule secreted by *A. viteae*, ES-62, which has possible therapeutic potential in autoimmune diseases such as Rheumatoid Arthritis (17). BMDCs exposed to ES-62 are capable of inducing a Th2 response. This capacity however, is not due to alterations in the cell surface markers, CD40, CD80, CD86 and CD54 (21). Furthermore, peritoneal macrophages produce low levels of IL-12, IL-6 and TNF-α following stimulation with ES-62, unlike LPS which elevates these cytokines dramatically. Interestingly, when ES-62 and LPS were cultured with the macrophages along with LPS, the levels of IL-12, IL-6 and TNF-α were suppressed to levels secreted by the addition of ES-62 alone, implying the importance of ES-62 in immune modulation.

The studies presented here use a very heterogeneous mix of isolate E/S antigens (7) rather than any defined antigen. Future work will involve fractionation of the S isolate E/S antigens, into high and low molecular weight components. This will begin to define the E/S products which provoke the macrophages and dendritic cells to secrete elevated levels of IL-10 and IL-6 and which may therefore contribute to parasite survival. In this context, B cell recognition of low molecular weight E/S antigens has previously been linked with a chronic infection outcome (7). Nevertheless, the S isolate total E/S is able to stimulate both BMMΦ and BMDCs to secrete significantly more IL-10 and IL-6 than E/S from either the J or E isolates. The majority of BMDC and BMMΦ used in this study had cell surface markers representative of dendritic cell or macrophage phenotype – BMDCs (*c.* 60% CD11c+CD11b+) and BMMΦ (*c.* 70% F4/80+CD11b+). It is possible therefore that some of the responding cell types may be immature bone marrow precursors. However despite this possibility, clear differences in the cytokine profiles are seen after exposure to the different isolate antigens.

The high levels of IL-10 and IL-6 secreted by these cells may underlie in part how the S isolate survives within the C57BL/6 host. Indeed, the induction of IL-10 and/or IL-6 *in vivo* and *in vitro* has been suggested to aid parasite survival or control host pathology (31–33). IL-10 is a regulatory cytokine (34). It can dampen-down both Th1 and Th2 responses (35) and has been shown to be important in the control of pathology during a *T. muris* infection (36). These data would suggest that the S isolate E/S has evolved to stimulate a more regulatory environment. In addition, data from our laboratory demonstrates that the S isolate, which survives in C57BL/6 mice, induces more regulatory T cells *in vivo* compared to the E isolate which is readily expelled (D’Elia *et* *al*. unpublished data).

The increase of IL-6 is slightly more puzzling, with the majority of IL-6 data focusing on the ability of IL-6, in conjunction with TGF-β, to induce Th17 cells. Further, IL-6 is known to inhibit the development of regulatory T cells (37). However the data here shows that BMMΦ secrete lower levels (similar to unstimulated) of TGF-β when stimulated with S isolate E/S compared to the other isolates. Therefore the balance of IL-6 and TGF-β may not be conducive for the generation of a Th17 response. There is contrasting data on the role of IL-6, with some research suggesting that IL-6 leads to the development of a Th2 response (38) or the dampening-down of a Th1 response (39). However, IL-6 has also been shown to be important in the up-regulation of IL-10 (40) and in this context may suggest that the S isolate is enhancing the regulatory environment via IL-6 production. Interestingly IL-6 deficient mice are still resistant to *T. muris* infection, implying that IL-6 is not important for the development of an effector Th2 response (Grencis *et* *al*. unpublished data).

Levels of IL-12p70 secreted by BMMΦ and BMDCs were not significantly elevated following S isolate E/S stimulation compared to the E and J isolate E/S, implying that the S isolate antigens are not simply promoting a stronger drive toward Th1 by inducing Th1-polarizing cytokines.

In conclusion E/S from the E, J and S isolates elevate IL-10 and IL-6 production from BMMΦ and BMDCs. Importantly these levels are significantly higher after exposure to the S isolate antigens compared to E or J isolate antigens.

It is difficult to relate the differences in APC- derived IL-10 and IL-6 seen here *in vitro*, to levels and differences which might be biologically significant *in vivo*. However, the differences in APC responses to the isolate antigens identified here have the potential to underlie the ability of the S isolate to survive successfully in the C57BL/6 host compared to the E and J isolates.

## Abbreviations

APCs: antigen presenting cells
BMMΦ: bone marrow derived macrophages
BMDCs: bone marrow derived dendritic cells
E/S: excretory/secretory antigen
